# Adipokine FABP4 integrates energy stores and counterregulatory metabolic responses

**DOI:** 10.1194/jlr.S091793

**Published:** 2019-01-30

**Authors:** Kacey J. Prentice, Jani Saksi, Gökhan S. Hotamisligil

**Affiliations:** Sabri Ülker Center for Metabolic Research* Harvard T. H. Chan School of Public Health, Boston, MA; Department of Genetics and Complex Diseases† Harvard T. H. Chan School of Public Health, Boston, MA; Broad Institute of Harvard and MIT§ Cambridge, MA

**Keywords:** obesity, metabolism, immunometabolism

## Abstract

Although counterregulatory hormones and mediators of the fight-or-flight responses are well defined at many levels, how energy stores per se are integrated into this system remains an enigmatic question. Recent years have seen the adipose tissue become a central focus for mediating intracellular signaling and communication through the release of a variety of bioactive lipids and substrates, as well as various adipokines. A critical integration node among these mediators and responses is controlled by FA binding protein 4 (FABP4), also known as adipocyte protein 2 (aP2), which is highly expressed in adipose tissue and functions as a lipid chaperone protein. Recently, it was demonstrated that FABP4 is a secreted hormone that has roles in maintaining glucose homeostasis, representing a key juncture facilitating communication between energy-storage systems and distant organs to respond to life-threatening situations. However, chronic engagement of FABP4 under conditions of immunometabolic stress, such as obesity, exacerbates a number of immunometabolic diseases, including diabetes, asthma, cancer, and atherosclerosis. In both preclinical mouse models and humans, levels of circulating FABP4 have been correlated with metabolic disease incidence, and reducing FABP4 levels or activity is associated with improved metabolic health. In this review, we will discuss the intriguing emerging biology of this protein, including potential therapeutic options for targeting circulating FABP4.

## Graphical Abstract

**Figure:**
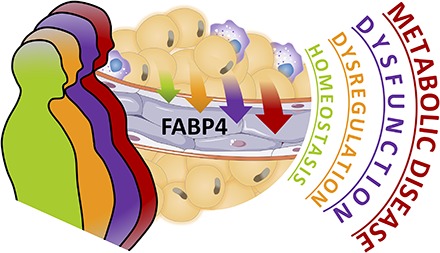

For the vast majority of human history, mankind has faced numerous challenges for survival, including scarce and unpredictable food supply, drought, predation, and exposure to pathogens. As such, various biological pathways have evolved to combat conditions of deprivation, orchestrating incremental responses to promote survival by mobilizing stored resources and adapting our metabolism to cope with such life-threatening circumstances. Dr. Walter Cannon first described the “fight-or-flight” response in the early 20th century in his study of the sympathetic nervous system and actions of adrenaline. These responses ensure that there is appropriate fuel supply in the form of glucose liberated from the liver to supply skeletal muscle and brain, enabling escape from predation, food scavenging, and alertness, in addition to numerous other biological effects. Similar adaptive defense systems can be envisioned to withstand starvation, hypoglycemia, and injury, which predominantly are engaged for a limited duration of time.

However, during recent human history, the Industrial Revolution has changed our world toward the plenty, contributing to an epidemic of obesity and chronic metabolic diseases. Noncommunicable diseases, particularly those of metabolic nature, account for more deaths worldwide than all of the most common infectious diseases combined (WHO). In a world of constant excess, the few adaptive mechanisms we have to combat what were previously temporary exposures to nutrient excess are consistently engaged, converting the “fight-or-flight” acute responses or short-term adaptive countermeasures into chronically engaged pathways to combat against prolonged exposure to excess nutrients and alterations in metabolism. This intersection of insufficient adaptive responses, combined with hyperengagement and chronic engagement of existing mechanisms, is a critical site for the development of targeted therapeutics that can potentiate our health and survival.

## DYSREGULATION OF FABP4 AS A CRITICAL MALADAPTIVE RESPONSE TO OBESITY

**Fig. 1.**
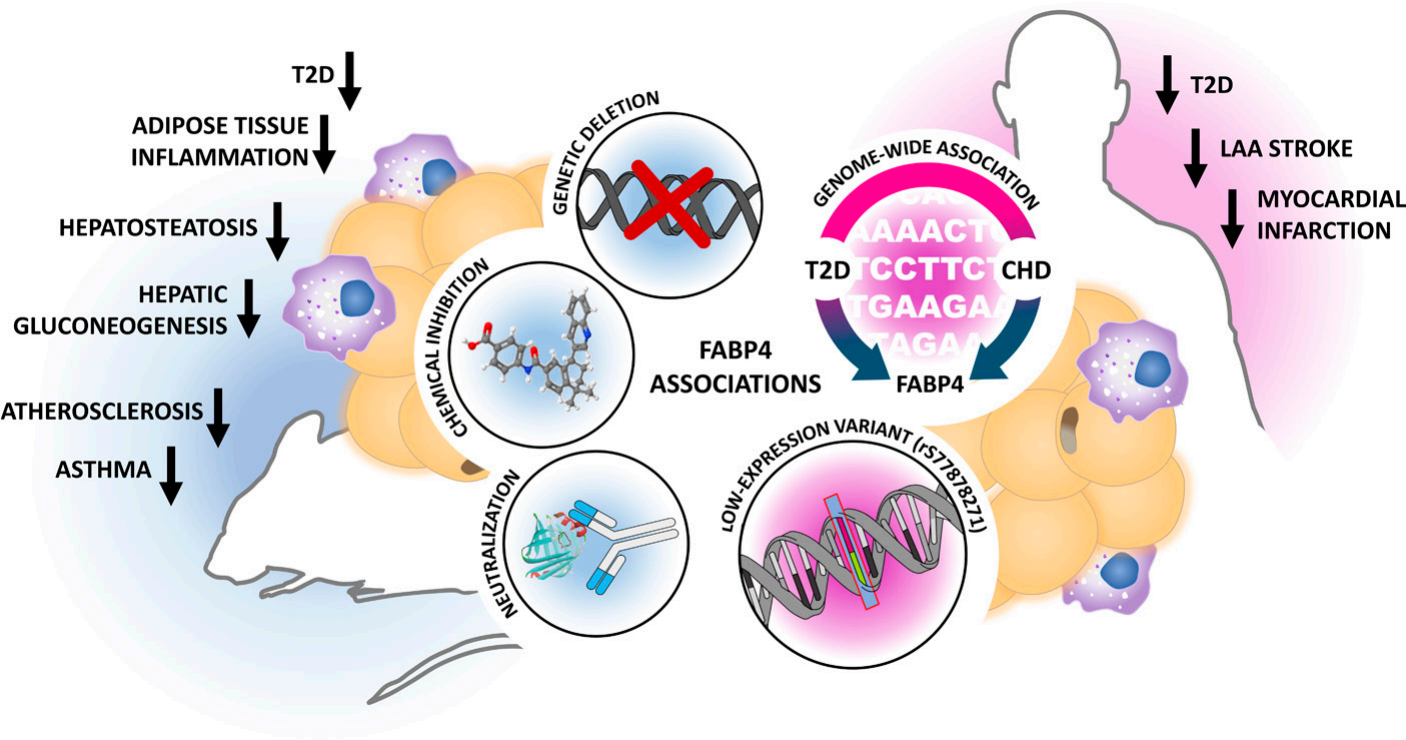
Evidence for a key role of FABP4/aP2 in immunometabolic diseases. Genetic FABP4/aP2 deficiency, inhibition through small molecules, or Ab-mediated targeting efficiently attenuates the development of various immunometabolic phenotypes in mice. In humans, FABP4 has been identified as a common candidate gene for the development of both T2D and CHD. Conversely, evidence from low-expression variant carriers suggests that reduced FABP4 gene activity is associated with improved lipid parameters and reduction in cardiometabolic endpoints in man. In the setting of genetic deficiency or genetically reduced expression of FABP4/aP2, many of the improved metabolic phenotypes are enhanced or exclusively evident in the context of obesity, suggesting that the systemic effects of obesity on immunometabolic risk may be mediated through the adipokine FABP4/aP2. LAA, large artery atherosclerotic.

A key component in survival responses is likely to reside in close proximity to energy stores, such as adipocytes. FA binding protein 4 (FABP4), also known as adipocyte protein 2 (aP2), may represent one such factor that is crucial for homeostasis and endurance, but maladapted to conditions of nutrient excess or chronic stress. Briefly, FABP4 is one of the most abundant proteins in adipocytes (1), with roles in maintaining adipocyte homeostasis, regulating lipolysis and adipogenesis through interactions with hormone-sensitive lipase (HSL) and peroxisome PPAR-γ, respectively (2, 3). Under conditions of lipolysis, such as fasting, FABP4 is suggested to bind FFAs within the cytoplasm, modulating the inhibitory activity of the liberated lipids on lipolytic enzymes (4) and contributing to their release from the cell. This response is beneficial for survival in the context of starvation, when distant tissues utilize lipids as an energy source. However, in obesity, where there is abundant adipose tissue, insulin resistance, and uncontrolled lipolysis, FABP4 is constantly engaged. This leads to harmful downstream effects in multiple tissue types, including the liver, cardiovascular system, and pancreatic β cells (5–8). Mice genetically lacking FABP4 (FABP4^−/−^) are almost completely protected against the development of various metabolic diseases, including diabetes, atherosclerosis, cancer, and asthma under distinct immunometabolic stress conditions, including diet-induced obesity, genetic obesity, or hypercholesterolemia (**Fig. 1**).

The high degree of evolutionary conservation of FABP4 from yeast to mammals implies that this protein likely has critical roles for survival (9). However, there has not yet been a human identified as genetically lacking FABP4. Although FABP4^−/−^ mice are viable and healthy (6), it is important to note that rodent models are only examined under controlled stresses such as high-fat diet, cold, or starvation, and there is more to be explored to understand FABP4 biology in humans. Despite this, independent groups have identified a rare low-expression variant of FABP4 in diverse human populations that is associated with protection against CVD and T2D (10, 11). This promoter mutation impairs C/EBP-α binding, reducing transcription and lowering FABP4 expression. Low-expression variant carriers thus exhibit lower incidence of T2D, reduced circulating triglycerides and cholesterol in the context of obesity, and reduced complications associated with atherosclerosis (Fig. 1). Complementary to these findings, a recent genome-wide association study of more than 500,000 individuals worldwide identified FABP4 as a common risk factor for both T2D and coronary heart disease (CHD) (12). Taken together, there is unequivocal evidence in humans that prevention of excess levels of FABP4 is metabolically highly beneficial.

Despite its initial characterization as an adipocyte FA binding protein, FABP4 has a diverse expression profile and distinct functions in numerous cell types, including macrophages, endothelial cells, and the bronchial epithelium. Within macrophages, FABP4 deficiency has been associated with beneficial effects on cholesterol trafficking, foam cell formation, inflammatory activation, and endoplasmic reticulum (ER) stress (7, 13–16). This has been shown to have pronounced effects on atherogenesis in the ApoE^−/−^ mouse model fed a Western diet, where bone marrow transplant studies have shown that macrophage FABP4 seems to be essential for the development of vascular lesions (14). Within the endothelium, FABP4 has been shown to be expressed specifically within capillaries and small veins of various tissues, including the liver, kidney, and pancreas (17). This is consistent with in vitro data in primary HUVECs, which have demonstrated that FABP4 is required for vascular endothelial growth factor signaling and cell proliferation, associated with angiogenesis (17, 18). This may have particular importance in the context of cancer, as FABP4 in obese individuals is associated with enhanced proliferative and migration capacity of cancer cells (19), contributing to metastasis and reduced survival (20). Together, these findings suggest that FABP4 governs diverse biology, depending on the tissue source and disease context. Furthermore, in essentially all of the contexts where FABP4 function has been examined, biological consequences have been observed in tissues that do not express FABP4, suggesting that signals or circulating molecules may be downstream of the function of this protein.

## HORMONAL FABP4

The recent discovery of hormonal FABP4 (5) created avenues of research focused not only in understanding how FABP4 is secreted, but also in how circulating levels correlate with disease and how FABP4 interacts with target cells to mediate biological activity. For the first time, it is possible to investigate previously perplexing questions regarding how deletion of FABP4 in distant tissues is implicated in the regulation of atherosclerosis formation, cancer progression, hepatic glucose production, and β-cell biology. Recent studies in models of atherosclerosis have demonstrated that local serum levels of FABP4 within the aorta correlate with disease severity (21). Transplantation studies of mammary tumor cells have shown that cells transferred into WT mice have greater proliferative potential than those transplanted into FABP4^−/−^ animals, despite tumor cells lacking FABP4 expression (19). Furthermore, FABP4^−/−^ animals exhibit a defect in β-adrenergic-stimulated insulin secretion, even under lean conditions (8), suggesting an effect on β-cell function, another cell type that does not express FABP4. This is complemented by human studies showing that higher serum FABP4 levels correlate with higher insulin response index in T2D patients and a higher insulinogenic index in nondiabetics (22). In all of these cases, the role of this hormonal FABP4 remains to be explored further.

Numerous studies have now also been conducted to examine the association between circulating FABP4 and various immunometabolic diseases in human populations (23). The strongest association with FABP4 is consistently BMI, with obese individuals having significantly higher circulating FABP4 levels than normal-weight controls (5). Interestingly, females exhibit higher circulating FABP4 levels than males, perhaps suggesting differences in adipose depot contributions and/or the involvement of sex hormones in the regulation of secretion (24). Circulating FABP4 levels have been independently correlated with increased risk for CVDs, early prediction of myocardial infarction (25), poor prognosis following stroke (26), T2D (27), nonalcoholic fatty liver disease (28), worsened glycemic control in type 1 diabetes (29), diabetic complications including retinopathy and nephropathy (30), preeclampsia (31), and association with various cancers, including breast cancer (19) and leukemia (20). Although these associations are robust, the mechanisms underlying how FABP4 contributes to disease pathogenesis remain enigmatic. This calls for new avenues of mechanistic research and creates paths for the development of therapeutic interventions that specifically target hormonal FABP4.

## SECRETION OF FABP4

**Fig. 2.**
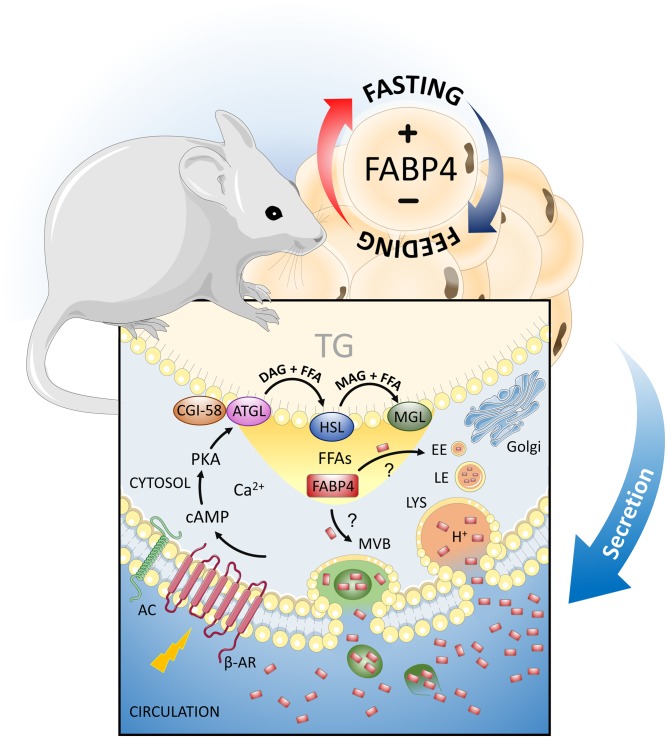
Regulation of FABP4/aP2 secretion from adipocytes. Circulating FABP4/aP2 levels are increased during obesity and regulated in response to feeding and fasting. In the fed state, insulin suppresses FABP4/aP2 secretion from adipocytes, whereas fasting and signals downstream of lipolytic stimuli induce secretion. Activation of the β-adrenergic receptor (β-AR) raises intracellular cAMP levels, which act to initiate lipolysis. Induction of FABP4/aP2 secretion requires the actions of lipolytic enzymes ATGL and HSL, and to a lesser extent MGL, and the liberation of FFAs. Once lipolysis is initiated, FABP4/aP2 may be released in a compartmentalized fashion through the lysosomal pathway, and to lesser extent through multivesicular bodies (MVBs). How FABP4/aP2 is recruited into these vesicular compartments remains to be explored. AC, adenylyl cyclase; CGI-58, comparative gene identification-58; DAG, diacylglycerol; EE, early endosome; LE, late endosome; LYS, lysosome; MAG, monoacylglycerol; PKA, protein kinase A; TG, triglyceride.

Importantly, most of the disease correlations with serum FABP4 levels are observed in the context of obesity, suggesting that adipocyte-derived FABP4 is likely to be the source of this pathogenic molecule. Mechanisms of FABP4 secretion from adipose tissue have been strongly correlated with signals downstream of lipolytic stimuli, which are consistently engaged during insulin resistance, stress, and obesity (**Fig. 2**). In vivo, FABP4 levels increase in response to fasting, sympathetic nervous system activation, or treatment with a β3-receptor agonist such as isoproterenol or CL-316423 and are suppressed under refeeding or insulin, consistent with this biology (5, 32, 33). Mechanistic studies have revealed that these signals potentiate FABP4 secretion through increases in intracellular cAMP and associated Ca^2+^ influx, as confirmed by the increase observed with forskolin treatment (34, 35). Furthermore, studies in adipocyte cell lines show that stimulation of FABP4 secretion is dependent on lipolytic machinery, with inhibition of more proximal proteins in the pathway such as adipose triglyceride lipase (ATGL) and HSL blunting FABP4 secretion to a greater degree than the distal component monoacylglycerol lipase (MGL) (32). This suggests that the liberation of FAs may be important for the secretion of FABP4. However, the exact mechanisms underlying the secretion of FABP4 remain to be explored, as it lacks a classical secretion peptide motif, and inhibitors of the classical secretion pathways do not alter the FABP4 secretion profile (36).

Intriguingly, FABP4 may be released in a compartmentalized fashion. A recent study demonstrated that FABP4 is secreted through the lysosomal pathway, as chloroquine, which acts by raising the luminal pH of lysosomes and preventing membrane fusion, was shown to reduce stimulated FABP4 secretion both in vitro and in vivo (37). This important study provides a compelling mechanism for the secretion of free FABP4 and defines a rare pathway utilized for nonclassical protein secretion in mammals. There is also literature suggesting that FABP4 secretion occurs, at least to some extent, through vesicular bodies. Immunogold electron microscopy has revealed the presence of a subset of FABP4 in multivesicular bodies contained within adipocytes (32), and reports have identified FABP4 on the surface of adipocyte-derived exosomes (38). However, it is unclear how FABP4 is specifically targeted to exosomes or is associated with other vesicular structures. It is interesting to note that, although this vesicular FABP4 accounts for only a minor fraction (∼0.5%) of the total secreted pool of FABP4, it is regulated in mouse and human obesity (32). Further understanding of potential pathways of FABP4 secretion (as summarized in Fig. 2), including regulation of trafficking of cytosolic FABP4 to vesicles, remains an important avenue of research.

## FACTORS INFLUENCING ACTIVITY OF HORMONAL FABP4

A fundamental aspect of FABP4 biology is in understanding how hormonal FABP4 influences target cells to regulate activity. In general, the phenotypes seen in FABP4 loss-of-function models are robust and very consistent. However, gain-of-function models are limited and more challenging, especially with the use of standard protocols to produce recombinant protein. This may be due to the amount of FABP4 needed, its rapid clearance from circulation, the timing of dosing, or, importantly, because we do not yet fully understand the differences between the recombinant and natural form(s) of this protein in circulation. Because FABP4 binds to multiple lipid ligands with similar affinities, a specialized endogenous lipid cargo could dictate its activity and function (39). BMS309403, a small-molecule inhibitor of FABP4 used in both in vivo and in vitro applications, functions by binding to the lipid-binding pocket of FABP4 and inhibiting interaction with at least some lipid cargo (40). Furthermore, a lipid-binding mutant form of FABP4 shows a similar lack of activity, suggesting that lipid binding may be essential to at least some aspects of FABP4 biology (5). The induction of FABP4 secretion in response to lipolysis and liberation of FFAs within adipocytes further supports the notion that FABP4 may exist in various functional forms, depending on its cargo or the conditions that trigger its release (32, 37). Expanding upon this idea, the cellular source of FABP4 may also have critical roles in dictating the extracellular function of the protein. Both macrophages and endothelial cells express and secrete FABP4, albeit at much lower levels per cell than adipocytes (41, 42). As both of these cell types contain relatively low levels of FFAs, and FABP4 is believed to play alternative roles in their intracellular biology, including regulation of ER stress and angiogenesis, respectively, it is possible that secreted FABP4 from these sources has differential physiological roles, which may not be appropriately modeled by recombinant protein.

It is also possible that FABP4 exists in multiple forms. Mass spectrometry-based screens have independently identified numerous posttranslational modifications on FABP4, including phosphorylation, acetylation, and carbonylation (43–45). However, only phosphorylation has been validated in in vitro systems, and the functional relevance of such modification(s) remains to be addressed (44). Finally, FABP4 can interact with numerous proteins, including HSL and cytokeratin 1, and with itself to potentially form oligomeric structures (2, 46, 47). Therefore, it is possible that FABP4 functions as part of a complex to mediate or fine tune its biological activity and that such complex component(s) may be critical for its diverse functions, as shown for other adipocyte-derived hormones, such as adiponectin (48). Therefore, the investigation of the natural FABP4 hormone and its potential pathological variations remains an essential and incompletely addressed topic in the field, one that will be critical for therapeutic technologies to target this protein.

## THERAPEUTIC TARGETING OF FABP4

Understanding that FABP4 functions hormonally has transformed the perspective on utilizing FABP4 as a therapeutic target for immunometabolic diseases. Historically, FABP4 has been targeted using four major approaches: constitutive genetic deletion, temporal genetic manipulation (siRNA, RNA interference, etc.), small-molecule inhibitors, and, much more recently, neutralizing Abs. Genetic deletion, either constitutive or through silencing approaches, has shown remarkable consistency in the improvement of metabolic phenotypes in multiple independent studies (5, 6, 8, 14, 15, 33). These studies have been complemented with small-molecule inhibitors, particularly BMS309403, but also with more potent related small molecules (49), which inhibit lipid binding and reduce the activity of FABP4 both intracellularly and presumably in circulation (40). These aspects require further clarification. It was only with the use of Ab therapies, however, that hormonal FABP4 was clearly identified as having a robust role in metabolic diseases, as Abs cannot enter cells and cannot pass the blood-brain barrier to act within the CNS (50). This exciting development allows us to understand the contribution of serum FABP4 to metabolic diseases in the context of peripheral tissues and develop a greater understanding of how targeting circulating FABP4 may be used clinically for the prevention or treatment of immunometabolic diseases.

## CONCLUSIONS

Overall, FABP4 has emerged as a critical player in immunometabolic diseases, with strong correlations between preclinical models and various human populations supporting the targeting of FABP4 for therapeutic benefit. As we look toward the development of FABP4-based therapies, we may need to first take a step back and refine/develop our understanding of the complex basic biology underlying this small FA-binding protein that resides in and out of the cell. Understanding the influence of different tissue sources, lipid cargo, posttranslational modifications, interacting partners, and regulation of secretory pathways will all be critical for developing potent therapeutics with the greatest disease relevance, as these factors may define relevance to distinct pathologies. Furthermore, in the development of therapeutics, we may also wish to consider the specific activity or system of interest to target. For instance, we may have to consider that immunometabolic responses regulated by FABP4 may also be mediated by its activity in the brain. For example, there is suggested to be a component of insulin resistance in neurodegeneration and lesion formation that may be influenced by FABP4, which could not be addressed by an Ab-based therapy. Thus, this drives the need for new generation of small-molecule inhibitors that may also target the brain.
